# Identification of proteins associated with the yeast mitochondrial RNA polymerase by tandem affinity purification

**DOI:** 10.1002/yea.1672

**Published:** 2009-08

**Authors:** Dmitriy A Markov, Maria Savkina, Michael Anikin, Mark Del Campo, Karen Ecker, Alan M Lambowitz, Jon P De Gnore, William T McAllister

**Affiliations:** 1 Departments of Cell Biology, University of Medicine and Dentistry of New Jersey, School of Osteopathic MedicineStratford, NJ, USA; 2 Graduate School of Biomedical Science, University of Medicine and Dentistry of New JerseyStratford, NJ, USA; 3 Institute for Cellular and Molecular Biology, Department of Chemistry and Biochemistry, and Section of Molecular Genetics and Microbiology, School of Biological Sciences, University of Texas at AustinTX, USA; 4 Summer Undergraduate Research Experience Program, University of Medicine and Dentistry of New JerseyStratford, NJ, USA; 5 Tufts University Core Facility, Department of Physiology, Tufts Medical SchoolBoston, MA, USA

**Keywords:** mitochondria, *Saccharomyces cerevisiae*, Rpo41p, RNA polymerase, transcription, tandem affinity purification, TAP-tag, Mss116p, DEAD-box protein

## Abstract

The abundance of mitochondrial (mt) transcripts varies under different conditions, and is thought to depend upon rates of transcription initiation, transcription termination/attenuation and RNA processing/degradation. The requirement to maintain the balance between RNA synthesis and processing may involve coordination between these processes; however, little is known about factors that regulate the activity of mtRNA polymerase (mtRNAP). Recent attempts to identify mtRNAP–protein interactions in yeast by means of a generalized tandem affinity purification (TAP) protocol were not successful, most likely because they involved a C-terminal mtRNAP–TAP fusion (which is incompatible with mtRNAP function) and because of the use of whole-cell solubilization protocols that did not preserve the integrity of mt protein complexes. Based upon the structure of T7 RNAP (to which mtRNAPs show high sequence similarity), we identified positions in yeast mtRNAP that allow insertion of a small affinity tag, confirmed the mature N-terminus, constructed a functional N-terminal TAP–mtRNAP fusion, pulled down associated proteins, and identified them by LC–MS–MS. Among the proteins found in the pull-down were a DEAD-box protein (Mss116p) and an RNA-binding protein (Pet127p). Previous genetic experiments suggested a role for these proteins in linking transcription and RNA degradation, in that a defect in the mt degradadosome could be suppressed by overexpression of either of these proteins or, independently, by mutations in either mtRNAP or its initiation factor Mtf1p. Further, we found that Mss116p inhibits transcription by mtRNAP *in vitro* in a steady-state reaction. Our results support the hypothesis that Mss116p and Pet127p are involved in modulation of mtRNAP activity. Copyright © 2009 John Wiley & Sons, Ltd.

## Introduction

Mitochondria are self-replicating organelles that possess their own genome and are thought to be derived from an ancient bacterial endosymbiont (Grey *et al.*, 1999). They are responsible for production of most energy in eukaryotic cells through the process of oxidative phosphorylation (Saraste, 1999). Reactive oxygen species (ROS) generated as a by-product of oxidative phosphorylation damage both cellular and organelle constituents (Cadenas and Davies, 2000; Huang and Manton, 2004), and it has been suggested that ROS-induced damage to mtDNA is a major reason for the loss of mt function during age-related pathologies (Hiona and Leeuwenburgh, 2008; Wei *et al.*, 1998). However, it has recently been found that mtRNA transcripts may be even more sensitive to oxidative damage than DNA (Li *et al.*, 2006), and it has been suggested that balancing synthesis and degradation of mt transcripts is required to preserve the quality of mtRNAs and organelle integrity (Rogowska *et al.*, 2006).

Our understanding of protein factors that modulate mitochondrial transcription or couple RNA synthesis with RNA processing and degradation is limited, and is derived primarily from genetic and biochemical studies in the budding yeast *Saccharomyces cerevisiae* (Calvo *et al.*, 2006; Shadel, 2004). In yeast (as well as in higher eukaryotes) mtRNA synthesis is driven by a small, single-subunit DNA-dependent RNA polymerase that is homologous to bacteriophage T7 RNA polymerase (Masters *et al.*, 1987). Only a limited number of genes have been identified as suppressors of mutations in the nuclear gene that encodes yeast mtRNAP (*RPO41*) and, with the exception of the initiation factor Mtf1p (Mangus *et al.*, 1994; Matsunaga and Jaehning, 2004), none of their products have been demonstrated to possess a definite function *in vitro*. These include the inner membrane protein Sls1p, which has been suggested to anchor the transcription complex to the inner mt membrane through direct interaction with the mtRNAP N-terminal domain (Bryan *et al.*, 2002; Rodeheffer and Shadel, 2003), and the matrix protein Nam1p (Mtf2p), which serves as a linker between mtRNAP and a pool of other protein factors, including Pet309p, Cbp1p and a large 900 kDa protein complex with still unknown composition (Shadel, 2004).

An earlier approach towards identifying factors associated with mtRNAP involved passage of human mt extracts over an affinity column having immobilized mtRNAP (Wang *et al.*, 2007). While this identified a ribosomal protein (L12) that stimulates mt transcription *in vitro* and suggested a mechanism that may coordinate mt transcription and translation, it did not uncover any known or putative transcription factors. Separate attempts to analyse the mt proteome by two-dimensional gel electrophoresis also did not provide additional information on the organization or composition of the mt transcription apparatus (Balaban, 2006; Reinders and Sickmann, 2007).

The above considerations called for an alternative means to identify proteins associated with mtRNAP. The use of a tandem affinity purification (TAP) protocol to isolate highly pure protein complexes, while minimizing procedures that disrupt the association of their components, has become increasingly popular for such applications (Collins and Choudhary, 2008; Puig *et al.*, 2001) and has resulted in the characterization of a number of protein complexes, including the discovery of 12 previously unidentified proteins associated with yeast mt ribosomes (Saveanu *et al.*, 2001). The potential of the TAP method as a proteomic tool led to the creation of a genome-wide yeast library (to date the only eukaryotic library in which all putative ORFs were tagged), allowing investigators to perform a comprehensive analysis of the yeast ‘interactome’ in an attempt to reveal the association of each TAP-tagged protein with its functional counterpart(s) (Collins *et al.*, 2007a, 2007b; Gavin *et al.*, 2002; Krogan *et al.*, 2006). Unfortunately, this approach did not provide information about the mtRNAP transcription apparatus. This lack of success was likely due to a number of factors. Perhaps most importantly, the yeast TAP library utilized C-terminal TAP tag fusions. It is known that alterations at the C-terminus disrupt the activity of T7-like RNAPs, and thus these strains would have lost functional mitochondria. TAP pull-downs of transcription factors known to be associated with Rpo41p by genetic or biochemical approaches (e.g. Mtf1p) also failed to confirm their association with the core mtRNAP (Table 1), possibly due to the use of whole cell extracts, in which the integrity of the mitochondrial nucleoid was not maintained (Gavin *et al.*, 2002), and the use of high-salt cell solubilization buffers that interfere with the stability of DNA–protein complexes and the activity of T7-like RNAPs (Winkley *et al.*, 1985; Maria Savkina, unpublished).

**Table 1:** Results of global proteome search for proteins associated with the mt transcription apparatus

Proteins^1^	Function^2^	Associated proteins by affinity capture-MS^3^	Associated proteins by genetic and biochemical methods^4^
**Rpo41p**	Mt RNAP similar to phage T7 RNAP; requires a specificity factor Mtf1p for promoter recognition	*No data*	Mtf1p, Mtf2p (Nam1p), Sls1p, Pet309p*
**Mtf1p**	Mt RNAP specificity factor	**Erg6p, Pzf1p, Ecm27p, Caf4p, Sub2p, Tor1p, Snf5p, Tfb1p**	**Rpo41p**
**Mtf2p (Nam 1p)**	Matrix protein coupling transcription, RNA processing, and translation	**Sls1p, Adh3p, Rvs161p, Skt5p, Urh1p**	**Rpo41p, Pet309p**
**Sls1p**	Inner membrane protein that coordinates the gene expression; may facilitate delivery of mRNA to translation machinery	**Mtf2p (Nam1p)**	**Mtf2p (Nam1p), Kar2p, Rpo41p**
Pet309p*	Inner membrane protein, specific translational activator and stabilizer for the *COX1* mRNA	**Cbp1p**	**Mtf2p (Nam1p)**

1 Proteins that are known to interact with the transcription apparatus and

2 their reported functions (Shadel, 2004).

3 Associated proteins identified by combination of affinity capture and mass spectrometry (MS) in a global screen of a library of *S. cerevisiae* strains with TAP-tagged ORFs [Collins *et al.*, 2007a, 2007b; Gavin *et al.*, 2002; Krogan *et al.*, 2006; data are available in the Biological General Repository for Interaction Datasets (BioGRID) database (http://www.thebiogrid.com)]. Proteins that have not been shown to have mt function are underlined.

4 Protein interactions established by genetic and biochemical methods.

* Pet309p does not interact with Rpo41p directly; instead, it is associated with the core transcription enzyme through Mtf2p (Nam1p).

By modifying the TAP pull-down method to take into account these considerations, we successfully identified a number of proteins that were previously known or, more importantly, previously unknown, to be associated with the yeast mt transcription apparatus. Our results support previous hypotheses involving the need to balance rates of RNA synthesis and degradation, and suggest mechanisms by which mtRNA synthesis and degradation may be coordinated.

## Materials and methods

### Yeast and bacterial strains, cloning, and mutagenesis

All reagents were purchased from Sigma-Aldrich unless otherwise specified. The heterozygotic diploid *Saccharomyces cerevisiae* yeast strain BY4743 (MAT**a**/MATα his3Δ1 leu2Δ0 met15Δ0 ura3Δ0) was purchased from Open Biosystems Inc. Yeast cells were cultured on YEP media with 1.5% agar at 30 °C unless otherwise specified. *E. coli* XL10-Gold competent cells (Stratagene) were used for all subcloning and site-directed mutagenesis procedures. Yeast DNA was isolated from strain BY4743 as described (Sambrook and Russell, 2001); a 5.8 kb DNA fragment encoding the *RPO41* gene (Masters *et al.*, 1987) was amplified from yeast chromosomal DNA using an Expand Long Template PCR System kit (Roche Applied Science), subcloned into pT7Blue vector, using a Perfectly Blunt cloning kit (EMD Bioscience/Novagen), and recloned into pRS316 and pRS315 yeast shuffling vectors (centromeric plasmids that exist as a single copy in the cell and thus ensure the lack of overexpression; Sikorski and Hieter, 1989) to produce pRS316–Rpo41p and pRS315–Rpo41p, respectively. Competent cells were prepared and transfected with the shuffling constructs as described in BD Yeastmaker Yeast Transformation System 2 User Manual (BD Biosciences). Yeast sporulation, dissection, and mating to confirm the haploid phenotype of the BY4743 derivative (MAT**a** his3Δ1 leu2Δ0 met15Δ0 ura3Δ0)::pRS316-Rpo41p were carried out by standard methods (Guthrie and Fink, 1991). Yeast containing shuffling constructs based on pRS316 and pRS315 vectors were selected on synthetic complete medium with Dextrose (SD) lacking either uracil or leucine, respectively, and prepared as described (Guthrie and Fink, 1991). To select against the pRS316–Rpo41p vector, 5-FOA medium was prepared by adding 5-FOA to SD medium at a concentration of 1 mg/ml (Zymo Research). *RPO41* insertion mutants encoding eight His residues downstream of Asp33, Glu390, Asn773, Gly858, M928, Ala1001, and the first 173 amino acids (aa) of a TAP tag from pMK33–NTAP vector (Veraksa *et al.*, 2005) downstream of Asp33 at Rpo41p ORF were constructed using a mega-primer extension method (Sarkar and Sommer, 1990) and standard molecular cloning manipulations on the background of the plasmid pRS315–Rpo41p described above. All plasmids encoding mutant and wild-type *RPO41* genes were purified using QiaQuick Miniprep kits (Qiagen) and submitted for DNA sequencing to Genewiz Inc.

### Isolation of yeast mitochondria

Isolation of a crude fraction of yeast mitochondria was performed as described (Pon and Schon, 2001), with slight modifications as noted below. Cells were grown in a modified rich medium previously used in studies of organelle transport in isolated mitochondria (Daum *et al.*, 1982; Suissa and Schatz, 1982) and contained the following ingredients: 1% peptone, 1% yeast extract, 2% galactose, 0.1% KH_2_PO_4_, 0.12% (NH_4_)_2_SO_4_, 0.05% CaCl_2_, 0.06% MgCl_2_, 0.0003% FeCl_3_, and 10 mg/l tetracycline. After vigorous agitation in 4 l baffled Erlenmeyer flasks with 10 drops of Antifoam Y-30, cells from mid-log 1.5 l culture (OD_600nm_ = 5.0) were collected by centrifugation, and spheroplasts were obtained by rocking cells at 30 °C for 45 min with crude lyophilized lyticase from *Arthrobacter luteus* (∼600 units/g cells). Mitochondria were recovered from spheroplasts by osmotic lysis, with the addition of Protease Inhibitor Cocktail for use with fungal and yeast extracts (Sigma-Aldrich) and several steps of differential centrifugation, as described (Pon and Schon, 2001), and were further purified by centrifugation through a sucrose step gradient as described (Meisinger *et al.*, 2000). After two washes with SEM buffer (Meisinger *et al.*, 2000), organelles were harvested at 12 000 × *g* for 10 min in 1.5 ml Eppendorf tubes. Pellets (∼200 µl/tube) were used immediately or flash-frozen in an acetone/dry ice mixture and stored at −70 °C for subsequent analysis.

### Isolation of mt nucleoids and *in vitro* transcription

Mt nucleoids were isolated as originally described (Miyakawa *et al.*, 1987), with additional modifications (Meeusen *et al.*, 1999; Miyakawa *et al.*, 2003). Purified nucleoids were recovered as a DAPI-positive interphase between 15% and 40% layers of a sucrose step gradient and resuspended in nucleoid buffer (20 mm Hepes–KOH, pH 7.6, 0.5 m sucrose, 20 mm KCl, 2 mm EDTA, 0.8 mm spermidine, 0.4 mm phenylmethanesulphonyl fluoride (PMSF), 7 mm β-mercaptoethanol). One µl nucleoid suspension was diluted into 10 µl transcription buffer (20 mm Hepes–KOH, pH 7.4, 25 mm sucrose, 20–200 mm KCl, 0.8 mm spermidine, 7 mm β-mercaptoethanol, 10 mm MgCl_2_, 300 µm CTP, GTP, ATP; 50 µm UTP; 1 mCi/ml α-^32^P-UTP) without addition of any external DNA template. Transcription reactions were carried out for 20 min at 30 °C, with or without addition of 10 mg/ml α-amanitin as indicated, and stopped by the addition of an equal volume of transcription stop buffer containing 90% formamide, 50 mm EDTA and 1 mg/ml bromophenol blue. After heating at 95 °C for 2 min, radioactively labelled RNA products were resolved by electrophoresis in polyacrylamide denaturing gels (Sambrook *et al.*, 1989) containing 12% acrylamide : bis-acrylamide mixture (19 : 1), 1× TBE buffer and 7 m urea, using a Model S2 Sequencing Gel Electrophoresys Apparatus (GibcoBRL) for 1.5 h at 90 W. Transcription rate was quantified as amount of radioactivity incorporated into high molecular weight RNA products, using a PhosphorImager Typhoon 9200 system (GE Healthcare) and ImageQuant software.

### Tandem affinity purification of protein from isolated yeast mitochondria

Tandem affinity purification was conducted based on principles originally described (Rigaut *et al.*, 1999), except for solubilization procedure and buffers, which were adopted from methods used for isolation of intact mitochondria nucleoprotein complexes (Meeusen *et al.*, 1999; Miyakawa *et al.*, 1987, 2003), as noted above. All steps were performed at 4 °C except for recovery of mitochondria (as noted).

200 µl frozen mt pellet were defrosted at 30 °C, washed once with 5 ml pre-warmed (30 °C) respiration buffer (RB) containing 10 mm Hepes–KOH, pH 7.4, 25 mm sucrose, 75 mm sorbitol, 100 mm KCl, 10 mm K_2_HPO_4_, 10 mm MgCl_2_, 2 mm ADP, 1 mm EDTA, 5 mm pyruvate, 5 mm malate, 1 mm glutamate, 1 mg/ml BSA, 50 µm dNTPs, 300 µm NTPs (adapted from Enriquez *et al.*, 1994, 1996; Micol *et al.*, 1997) and then resuspended in pre-warmed RB at 2 mg/ml of mitochondrial protein (1 mg is ∼50 µl of mt pellet). After gentle agitation in 15 ml culture tubes for 30 min at 30 °C to allow organelles to respirate and restore their metabolic activity, mitochondria were transferred to Eppendorf tubes, harvested by centrifugation (12 000 × *g* for 5 min) and immediately resuspended in 3 ml ice-cold solubilization buffer (20 mm Hepes–KOH, pH 7.4, 250 mm sucrose, 200 mm KCl, 1 mm EDTA, 1 mm spermidine, 7 mm β-mercaptoethanol, 0.2 mm PMSF). 75 µl 20% NP-40 were added slowly, drop-by-drop, to the mt suspension to a final concentration of 0.5%. After 15 min of incubation on ice, each lysate was split into two 1.5 ml tubes and non-soluble components were removed by centrifugation at 20 000 × *g* for 10 min. After centrifugation was repeated, the cleared supernatant was transferred into 1.5 ml non-stick microtubes (Phenix Research Products Inc.), which contained 50 µl pre-washed Protein A Sepharose 4B FF beads (Sigma-Aldrich). After 1 h of incubation at slow rotation to prevent bead sedimentation, the supernatant was separated from the beads on a touch-spin table microfuge (1000 × *g* maximum), using Micro Bio-Spin columns (Bio-Rad), and transferred to a fresh non-stick tube with 25 µl pre-washed IgG Sepharose 6 FF beads (GE-Healthcare/Amersham Bioscience). After 3 h rotation the supernatant was discarded and the beads were washed five times with 1.5 ml solubilization buffer containing 0.1% NP-40 and lacking EDTA (‘wash buffer’). Beads from two 1.5 ml tubes were combined in 0.6 ml non-stick microtubes (Phenix Research Products Inc.), resuspended in 500 µl wash buffer containing 10 µl AcTEV protease (100 U; Invitrogen) and rotation was continued for 6 h. The eluate was separated from the Sepharose beads on the Micro Bio-Spin columns and transferred to a fresh 0.5 ml tube containing 25 µl Calmodulin–Sepharose 4B beads (GE Healthcare) that had been pre-washed with wash buffer containing 2 mm CaCl_2_. After 3 h rotation, the beads were settled, washed five times with 0.5 ml wash buffer with 2 mm CaCl_2_, and twice with CaCl_2_-free buffer. Protein was eluted from the beads with three consecutive washes, each using 50 µl wash buffer with 2 mm EGTA, on ice for 15 min. The washes were combined, polypeptides were precipitated with acetone/TCA at −20 °C for 3 h and separated by electrophoresis in 4–12% Bis–Tris NuPAGE Novex protein gels in an MES buffer system (Invitrogen). The gels were stained with Coomassie Brilliant Blue R-250 (Bio-Rad), and each lane containing visible protein bands was sliced into 18 equal gel samples, which were submitted for tryptic digestion and LC–MS–MS analysis, as described below.

### LC–MS–MS analysis

Excised bands were subjected to in-gel reduction, alkylation and enzymatic digestion (Roche Applied Science, Indianapolis, IN, USA) in a HEPA-filtered hood to reduce the keratin background. LC–MS–MS analysis was performed on the in-gel digest extracts, using an Agilent 1100 binary pump (Santa Clara, CA, USA) directly coupled to a mass spectrometer. 2–8 µl of sample was injected onto the column using a LC Packings FAMOS autosampler (Sunnyvale, CA, USA). Nanobore electrospray columns were constructed from a 360 µm o.d., 75 µm i.d. fused silica capillary with the column tip tapered to a 10 µm opening (New Objective, Woburn, MA, USA). The columns were packed with 200 Å 5 µm C18 beads (Michrom BioResources, Auburn, CA, USA), a reverse-phase packing material, to a length of 10 cm. The pre-column was used to achieve a flow rate of 320 nl/min. The mobile phase used for gradient elution consisted of (a) 0.3% acetic acid and 99.7% water, and (b) 0.3% acetic acid and 99.7% acetonitrile. Tandem mass spectra (LC–MS–MS) were acquired on a Thermo Finnigan LTQ ion trap mass spectrometer (Thermo Corp., San Jose, CA, USA). Needle voltage was set to 3 kV, isolation width was 3 Da, relative collision energy was 30% and dynamic exclusion was used to exclude recurring ions. Ion signals above a predetermined threshold automatically triggered the instrument to switch from MS to MS–MS mode for generating fragmentation spectra. The MS–MS spectra were searched against the NCBI non-redundant protein sequence database, using the SEQUEST computer algorithm (Sadygov *et al.*, 2004), to produce a list of proteins identified in each sample. Allowance for a single oxidized Met was assumed, as protein oxidation occurs due to exposure to air, and during electrospray at 3 kV, etc. Taking into consideration a number of parameters, including the total number of peptides matched, the mass error on the match and the quality of match, protein identifications were accepted only when at least four and eight peptides were identified for each protein during non-redundant and informed protein searches, respectively, by Proteomic Browser software (Thermo, Waltham, MA, USA). We routinely observed a large number of polypeptides, typically more than 10, for each of the confident protein identifications made.

### Isolation of Rpo41p, Mtf1p and Mss116p

A plasmid that expresses N-terminally 6His-tagged Mtf1p (pMS34) was constructed following a previously reported approach (Schubot *et al.*, 2001). Briefly, yeast chromosomal DNA was purified as described (Sambrook and Russell, 2001) and utilized as a template to amplify the MTF1 ORF, using Vent DNA polymerase (NEB) and two oligonucleotides; the upstream primer contained an *Nco*I restriction site with an ATG codon followed by Gly6His-coding sequence, the downstream primer contained an *Xho*I site. The PCR product was cloned into a pTrcHisC expression vector (Invitrogen) to give pMS34. To express N-terminally 6His-tagged Rpo41p, the expression plasmid pMS11 was created as previously described (Matsunaga *et al.*, 2004). Amplified RPO41 ORF was inserted into pProEX HTb vector (Invitrogen) using *Stu*I and *Sac*I restriction sites. Both resulting plasmids were subjected to DNA sequencing in order to confirm the integrity of target constructs.

To purify His-tagged Mtf1p, *E. coli* BL21(DE3) codon plus RIPL cells (Stratagene) carrying the pMS34 plasmid were vigorously aerated at 20 °C in 0.5 l LB Miller broth supplemented with 0.1 g/l ampicillin until the culture reached an optical density of 0.6 at 600 nm. After addition of 0.1 mm IPTG followed by a further 8 h of aeration, cells were collected by centrifugation (6000 × *g* for 10 min), resuspended in 25 ml Mtf1p lysis buffer (50 mm sodium phosphate, pH 7.6, 300 mm NaCl, 3 mm β-mercaptoethanol, 10 mm imidazole, 1 mm PMSF), and disrupted on a Sonic Dismembrator 500 (Fisher Scientific), using 30 s pulses alternated with 30 s pauses over a total disintegration time of 20 min. The cell lysate was clarified by centrifugation (25 000 × *g* for 15 min) at 4 °C and applied to a 0.2 ml HisPur™ Cobalt Spin Column (Pierce) at room temperature (RT). The column was washed with 10 ml lysis buffer, and Mtf1p was eluted with 3 ml lysis buffer containing 150 mm imidazole. 1 ml fractions of eluate were collected, and the fraction containing the largest amount of protein was dialysed overnight at 4 °C against 500 ml dialysis buffer (40 mm Tris–HCl, pH 7.9, 300 mm NaCl, 5 mm β-mercaptoethanol and 5% glycerol). The dialysed protein was further purified using 1 ml HiTrap™ Heparin HP column (GE Healthcare) and a 10 ml gradient of 0.3–1.0 m NaCl in dialysis buffer on an AKTA HPLC system (GE Healthcare). The peak 1 ml fraction(s) corresponding to 0.42 m NaCl were pooled, diluted two-fold with glycerol and stored at −20 °C. Typically, the resulting solution contained ∼1 mg His-tagged Mtf1p at a concentration of 0.5 mg/ml, as determined by UV spectroscopy (OD of 1.8 at 280 nm corresponded to 1 mg/ml concentration of Mtf1p).

To purify His-tagged Rpo41p, *E. coli* BL21 codon plus RIL cells (Stratagene) containing pMS11 were grown at 25 °C in 0.5 l LB Miller broth supplemented with 0.1 g/l ampicillin until the culture reached an optical density of 0.5 at 600 nm. After the addition of 0.6 mm IPTG followed by a further 4 h of vigorous aeration, cells were collected by centrifugation, resuspended in 25 ml Rpo41p lysis buffer (40 mm Tris–HCl, pH 7.9, 300 mm NaCl, 0.1% Tween 20, 5 mm β-mercaptoethanol, 1 mm PMSF) and disrupted on a Sonic Dismembrator 500 (Fisher Scientific), as described for Mtf1p. The cell lysate was clarified by centrifugation as described, and NaCl was added to a final concentration of 0.8 m. 0.7 ml 10% poly(ethyleneimine) solution was added slowly, drop-by-drop, to the stirred lysate, and the precipitate was removed by centrifugation (10 000 × *g* for 5 min) at 4 °C following a 10 min still incubation on ice. The supernatant was diluted two-fold with a saturated solution of (NH_4_)_2_SO_4_ and incubated for 30 min on ice. The precipitate was collected by centrifugation (10 000 × *g* for 5 min), dissolved in 10 ml lysis buffer at 4 °C and filtered through a Poly-Prep Chromatography Column (Bio-Rad) packed with 0.5 ml Ni-NTA agarose (Qiagen) at RT. The column was washed with 5 ml lysis buffer followed by 5 ml lysis buffer containing 15 mm imidazole. The protein was eluted with 3 ml elution buffer (40 mm Tris–HCl, pH 7.9, 200 mm NaCl, 0.1% Tween-20, 5 mm β-mercaptoethanol, 150 mm imidazole, 1 mm PMSF). 1 ml fractions of eluate were collected, the fraction containing the largest amount of protein was loaded directly on a 1 ml HiTrap™ Heparin HP column (GE Healthcare), and the protein was eluted from the column with a 10 ml gradient of 0.2–1.0 m NaCl in buffer containing 40 mm Tris–HCl, pH 7.9, 5 mM β-mercaptoethanol, 5% glycerol. The peak 1 ml fraction(s) corresponding to 0.6 m NaCl were combined, distributed into aliquots and stored frozen at −70 °C. The typical yield of purified His-tagged Rpo41p was 3 mg at a concentration of 1.5 mg/ml, as determined by UV spectroscopy (1 mg/ml His-tagged Rpo41p corresponded to OD of 0.99 at 280 nm).

Mss116p was expressed and purified as previously described (Halls *et al.*, 2007) and stored in 10 µl aliquots at −70 °C in storage buffer (20 mm Tris–HCl, pH 7.5, 500 mm KCl, 1 mm EDTA, 1 mm DTT, 50% glycerol) at 5 µm concentration.

### Rpo41p *in vitro* transcription assay

Steady-state transcription reactions were carried out in a volume of 10 µl containing 20 mm Tris–HCl, pH 7.9, 50 mm KCl, 10 mm MgCl_2_, 0.1% Tween 20, 5 mm Tris (2-carboxyethyl) phosphine (TCEP; Thermo Fisher Scientific), 7 nm double-stranded DNA template (Figure 4A), 0.5 mm nucleotide 5′-triphosphates, 0.625 µm (α-^32^P-UTP (800 Ci/mm, Perkin-Elmer), 20 nm Rpo41p, 20 nm Mtf1p, and Mss116p as indicated (in 0.8 µl Mss116p storage buffer) at 30 °C for various times, and stopped with an equal volume of transcription stop buffer as described above. Labelled RNA products were resolved by electrophoresis on denaturing 15% polyacrylamide denaturing gels for 2 h, as described above.

## Results

### Identification of the mature N-terminus of yeast mtRNAP and construction of an N-terminal TAP fusion protein

**Figure 1:**
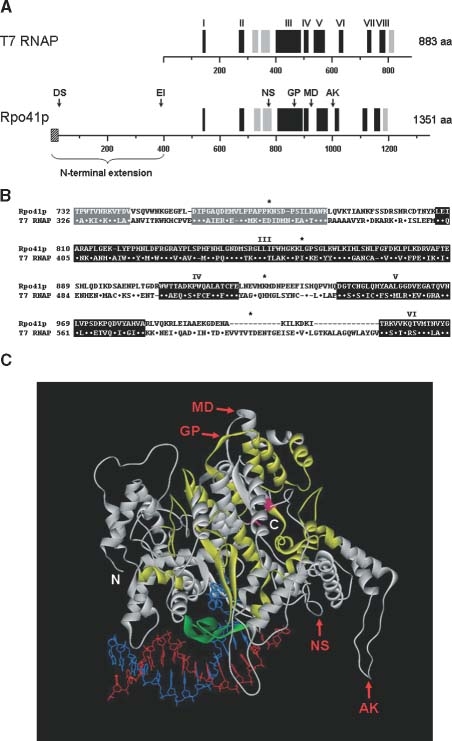
Conserved regions in Rpo41p and T7 RNAP. (A) Sequence alignment of Rpo41p and T7 RNAP. Vertical black bars represent eight highly conserved regions between Rpo41p and T7 RNAP (T7 RNAP aa 316–337, 351–378, 802–817; Masters *et al.*, 1987); grey bars correspond to additional regions with limited similarity. The width of the bars and the distances between them are scaled to the primary sequences of the corresponding proteins. Arrows indicate the relative positions of His-tag insertions and the names of corresponding mutants. The striped box at the N-terminus of Rpo41p indicates the relative size and position of the predicted mt targeting peptide (Wang and Shadel, 1999). (B) Alignments of targeted regions in Rpo41p. Sequences within regions of high conservation (black background) and limited similarity (grey background) are numbered as previously described. Amino acid residues are shown in one-letter code; dots represent identical residues; dashes indicate possible deletions. Pairs of residues between which His-tag insertions were introduced are indicated with asterisks. (C) Positions of His-tag insertions mapped onto the T7 RNAP-promoter structure (modified from PDB *1CEZ*; Cheetham *et al.*, 1999). The α carbon backbone of the T7 RNAP is represented as a ribbon cartoon; segments in yellow indicate regions that are highly conserved in Rpo41p (aa 142–150, 270–286, 402–483, 501–517, 537–575, 626–640, 725–740, 771–790); the region in green corresponds to the specificity loop (aa 739–770) that is responsible for promoter recognition (Temiakov *et al.*, 2000). DNA template and non-template strands are red and blue, respectively. Aspartic acid residues 537 and 812, which coordinate Mg^2+^ ions at the active centre and are conserved among all T7-like RNAPs, are shown in magenta. Positions where His-tags were inserted in the corresponding regions of Rpo41p are indicated with red arrows and the name of the mutant. N- and C-termini are marked with white letters; note that the highly conserved C-terminus is located within or near the active site

Previous studies have shown that whereas alterations in the C-terminal region of T7-like RNAPs (Figure 1C) result in inactivation of the enzyme (Gardner *et al.*, 1997; Mookhtiar *et al.*, 1991; Patra *et al.*, 1992), modifications to the N-terminus are well tolerated (He *et al.*, 1997; Pomerantz *et al.*, 2005). This suggested that it should be possible to construct an N-terminal TAP fusion of mtRNAP that would retain function. Like other nuclear-encoded mt proteins, Rpo41p is translated in the cytoplasm and delivered to the mitochondria by means of mt membrane translocases that recognize an N-terminal mt targeting peptide (Koehler *et al.*, 1999). The targeting peptide is subsequently cleaved by mt processing peptidases (MPP) to produce the mature N-terminus of the protein (Gakh *et al.*, 2002). To construct an N-terminal TAP-Rpo41p fusion, it was therefore first necessary to determine the natural N-terminus of the mature (processed) mtRNAP.

Although mt targeting peptides and their cleavage sites have been successfully predicted by similarity to known consensus motifs in a number of proteins (Fujiwara *et al.*, 1997; Kitada *et al.*, 2003), the reliability of such predictions is limited because MPP recognize many leader peptides with different primary and secondary structures, and can function at sites as far away as 100 residues from the N-terminus of the precursor protein (Graf *et al.*, 1988; Ito, 1999). In previous work it had been suggested that the first 29 N-terminal aa residues in Rpo41p are likely to comprise a mt targeting sequence (Masters *et al.*, 1987), and it was shown that fusion of this signal to various N-terminal deletion variants of Rpo41p was sufficient to direct delivery of the fusion variants to the mitochondria (Wang and Shadel, 1999). However, none of these deletion mutants (which affected regions downstream of the putative 29 aa signal peptide) were demonstrated to be fully functional *in vivo*, as even the smallest deletion resulted in a *ts*-petite phenotype and thus appeared to disrupt Rpo41p function (Wang and Shadel, 1999). The nature of the authentic N-terminus of the mature Rpo41p was therefore unclear.

A straightforward approach to identify the processed N-terminus of mtRNAP would be to isolate the mature polypeptide from organelles using affinity chromatography and to characterize it by Edman degradation. However, neither terminus of the Rpo41p precursor protein is suitable for modifications that would allow the insertion of an affinity tag, because adding a tag to the C-terminus (which forms part of the active site) would abolish enzyme activity and result in loss of functional mitochondria, while an N-terminal tag might block delivery of Rpo41p to mitochondria or would be cleaved off with the targeting peptide. To circumvent this problem, we sought to identify internal locations in Rpo41p that would tolerate small affinity tags, e.g. an eight aa histidine (His) tag, without disrupting function, and would be solvent-exposed.

Structural analysis of T7 RNAP reveals many surface exposed loops, and a number of these have been shown to tolerate substantial changes without disrupting function. Some of these regions are conserved in Rpo41p, suggesting that substitutions in these sites would also be tolerated by the mtRNAP (Figure 1C). Six potential positions in Rpo41p were chosen, all of which, based on the similarity of mtRNAPs to T7 RNAP, are thought to be on the surface of the enzyme (Figure 1A–C). An additional site that lies within the extended N-terminal domain of mtRNAP that is not present in T7 RNAP was also chosen (EI, Figure 1A, C; Wang and Shadel, 1999).

**Figure 2:**
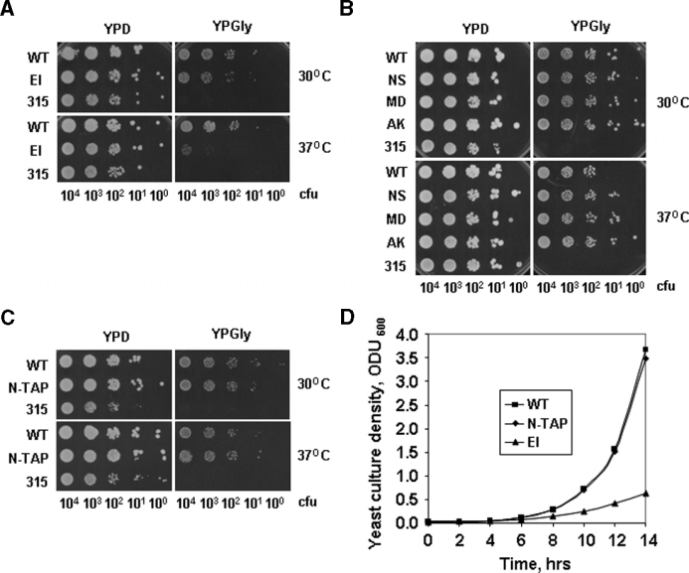
Respiratory activity of insertion mutants of Rpo41p *in vivo.* The chromosomal *RPO41* gene was replaced with a number of mutant genes containing insertions by using a plasmid-shuffling technique (see Materials and methods), and the ability of the resulting strains to respire was determined by plating on glycerol-containing media (YPGly) under normal (30 °C) and heat-shock conditions (37 °C) (YPD, YP w/dextrose; YPGly, YP w/glycerol; CFU, colony-forming units at appropriate dilutions). ‘315’ indicates a respiratory-defective strain (ρ^0^) used as control. Respiratory activities of EI (A), other His-tag insertion mutants (B) and N-terminal Rpo41p–TAP fusion mutant (N-TAP) (C) were determined in a similar manner. Strains expressing plasmid-encoded N-TAP-tagged and WT Rpo41p exhibited no difference in growth in a liquid medium with a non-fermentable carbon source (YP/galactose), while a strain harbouring a His-tag insertion in an alternative position (EI) did (D)

The resulting mutant mtRNAPs were substituted for the wild-type protein by a plasmid-shuffling technique. With the exception of mutant EI (which exhibited a ts-petite phenotype, Figure 2A), all derivatives supported growth on non-fermentable carbon sources under both optimal and heat-shock conditions, indicating that they were fully functional (Figure 2B). One of these mutants (MD, Figure 1B) was used to pull down the mature form of Rpo41p by metal affinity chromatography, and this protein was analysed by Edman degradation. Identification of the first five N-terminal aa of the mature, processed enzyme (ProSerProAspSer) confirmed the cleavage site that had previously been suggested (Masters *et al.*, 1987; Wang and Shadel, 1999).

Next, we determined whether affinity tags might be inserted just downstream of the processing/cleavage site without disrupting Rpo41p activity. To accomplish this, we constructed additional derivatives in which either a small eight aa His-tag or a large 173 aa TAP tag were inserted downstream of Asp33, immediately after the targeting peptide sequence (position DS, Figure 1A). The resulting proteins were functional, as the corresponding strains demonstrated no respiratory defects (see Figure 2C, D; data shown for TAP-tagged Rpo41p only).

### Tandem affinity purification and identification of Rpo41–TAP-associated proteins

Previous global proteomic studies in yeast involved a whole cell approach, using conditions that are unlikely to preserve the organization of mt nucleoids, and this may have contributed to the limited success of these studies in characterizing mt transcription complexes. We therefore sought to use methods that would preserve the integrity of the mitochondria and their nucleoids during the early stages of cell disruption and solubilization. It is known that, under proper conditions, isolated mitochondria retain their membrane potential and their transcription and replication activities for hours (Enriquez *et al.*, 1994, 1996; Micol *et al.*, 1997), and purified organelles have been used to isolate and characterize nucleoid preparations that can incorporate nucleotides into RNA (Hillar *et al.*, 1979).

**Figure 3:**
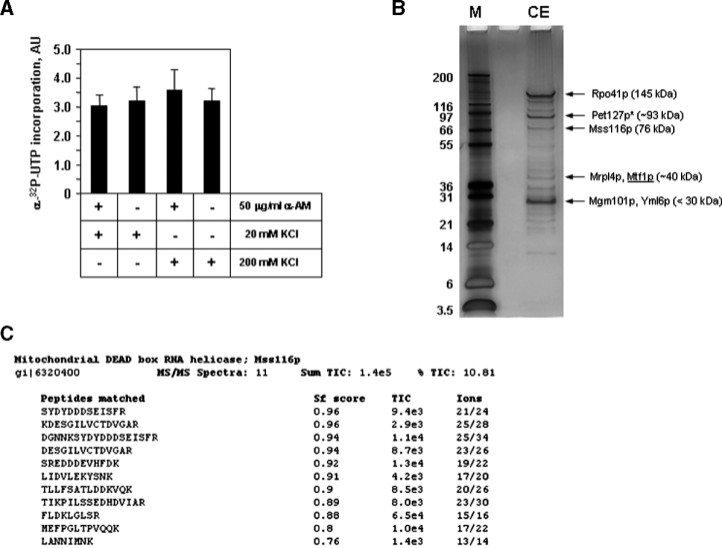
Isolation of active mt nucleoids and identification of proteins associated with TAP–mtRNAP. (A) Purification of transcriptionally active mt nucleoids. Nucleoids were purified as described (see Materials and methods) and diluted as indicated. Transcription reactions were carried out for 20 min at 30 °C in the presence of 20 or 200 mm KCl or α-amanitin (AM). The labelled RNA products were resolved by electrophoresis in a 12% acrylamide denaturing gel, visualized by autoradiography and quantified by PhosphorImager analysis. The abundance of heterogeneous high molecular weight products under various conditions is shown; error bars represent standard error for mt nucleoid transcripts (*n* = 3). (B) Rpo41p–TAP-associated proteins. Peptides eluted from the calmodulin–sepharose column were resolved on 4–12% bis-Tris NuPAGE Novex protein gels (Invitrogen) and visualized by silver staining (lane CE). Numbers indicate the molecular weights of protein markers in kDa (lane M). The bands from lane CE were submitted for LC–MS–MS analysis (Tufts University Core Facility). Arrows indicate silver-stained bands that match the molecular weight (MW) of major proteins identified in the gel slabs containing the bands. *Note that the actual MW of mature Pet127p may be smaller than predicted from its ORF as ∼90 N-terminal aa are likely to be absent in the mature form of Pet127p (Wiesenberger and Fox, 1997). (C) Example of LC–MS–MS spectra (Mss116p). Eleven peptides matching the Mss116p protein sequence were found as a result of an uninformed search against the *S. cerevisiae* protein database. S_f_, quality of match; TIC, total ion current; % TIC, signal or ion current for a given peptide; Sum TIC, sum of all TIC values for that protein identification—this exceeded the minimum required score in all cases. Additional information may be found at http://www.tucf.org. Complete LC–MS–MS scans are available upon request

As starting material for our TAP pull-downs, we isolated mitochondria under conditions that are optimized for *in organello* replication/transcription assays (Enriquez *et al.*, 1994, 1996; Micol *et al.*, 1997). Importantly, we found that transcription activity in the isolated mt nucleoid fraction is resistant to 200 mm KCl (Figure 3A), allowing the use of these conditions during subsequent isolation steps and thereby decreasing non-specific protein–protein interactions without disruption of salt-sensitive DNA–protein interactions that would occur at higher (500 mm) salt conditions, which are commonly used. To rule out the possibility that the observed salt-resistant transcription activity might be due to contaminating nuclear RNAPs, control reactions were carried out in the presence of α-amanitin, a potent inhibitor of nuclear RNAPs (Kelly and Lehman, 1986; Tsai *et al.*, 1971), which had no effect on transcription (Figure 3A).

After two affinity chromatography steps, proteins from the TAP elution were resolved by electrophoresis and visualized by silver staining. Eighteen gel samples containing ∼25 visible bands (Figure 3B) were excised and subjected to in-gel tryptic digestion and LC–MS–MS analysis. An example of LC–MS–MS spectra for one of the proteins identified (Mss116p) is shown in Figure 3C. Only proteins for which more than four distinct peptides were identified were considered significant (see Table 2). To eliminate consideration of proteins that might have bound non-specifically to the affinity column, negative control samples from cells that contained a shuffle vector that encoded Rpo41p which lacked the TAP tag were analysed in the same manner. Only a few proteins were identified in the control samples, including Atp1p (a very abundant subunit of mitochondrial ATP-synthase), and most of these were represented by less than four peptides. Importantly, none of these proteins was found among the peptides identified in the TAP-tagged Rpo41p pull-down (data not shown).

**Table 2:** Proteins identified in Rpo41p-TAP pull-down

Protein^1^	Number of peptides detected^2^	MW, kDa	Suggested function
**Pet127p**	**19**	**93**	mt matrix protein involved in RNA stability and processing; involved in transcriptional polarity
**Mss116p**	**11**	**76**	DEAD-box protein required for efficient splicing of mt introns
**Mrp14p**	**7**	**40**	mt ribosomal protein of the large subunit
**Kar2p**	**7**	**75**	ER protein import into the ER and protein folding; regulation of the unfolded protein response via interaction with Ire1p
**Ssc1p**	**7**	**71**	mt matrix protein involved in protein import; subunit of Scelp endonuclease; homolog of Hsp 70p
**Hsp60p**	**6**	**60**	mt chaperonin required for folding of precursor polypeptides and complex assembly; similar to *E. coli* GroEL
**YmI6p**	**7**	**32**	mt protein of the large ribosomal subunit similar to human MRP-L4 protein, deletion leads to petité phenotype
**Mgm101p**	**5**	**30**	component of the mt nucleoid required for the repair of oxidative mt DNA damage
**Pet309p**	**16**	**64**	specific translational activator for the *COX1* mRNA, also influences stability of intron-containing *COX1* primary transcripts; located in the mt inner membrane
**Mtf2p (Nam1p)**	**11**	**51**	mt matrix protein that interacts with mtRNAP and couples RNA processing, translation and transcription
**Sls1p**	**9**	**73**	mt inner membrane protein that coordinates gene expression; facilitates delivery of mRNA to membrane-bound translation machinery
**Mtf1p**	**5**	**40**	mtRNAP specificity factor with structural similarity to S-adenosylmethionine-dependent methyltransferases, interacts with mitochondrial core polymerase Rpo41p

1 Proteins were identified by LC–MS–MS analysis of gel-resolved polypeptides co-eluted with Rpo41p-TAP from calmodulin–Sepharose.

2 Only proteins having more than four identified peptides were considered positive (see Materials and methods); proteins identified as a result of an informed search for peptides having more than two oxidized methionines and processed by non-tryptic cleavage (see Materials and methods) are underlined.

The number of proteins found to be associated with the TAP-tagged mtRNAP was quite limited (Table 2), demonstrating the specificity of the pull-down. Nearly all of these proteins were known to be associated with mitochondria, yet none had previously been shown to be associated with the mt transcription apparatus. Surprisingly, none of the proteins previously thought to be associated with Rpo41p (i.e. Mtf1p, Mtf2p, Sls1p, Pet309p) (Shadel, 2004) was detected in this initial analysis. A potential reason for this is that these proteins may have been oxidized and, despite their association with Rpo41p, would have been excluded from the initial, non-redundant search (which discriminated against protein fragments that were oxidized and/or originated from denatured protein). In agreement with this, when we performed an informed search for polypeptides from these proteins that might contain oxidized aa residues and/or have a non-tryptic origin, we identified all proteins previously suggested to be associated with Rpo41p (i.e. Mtf1p, Mtf2p, Sls1p, and Pet309p; Shadel, 2004; Table 2). To exclude the possibility that the presence of the latter proteins was due to non-specific interactions with matrices, we performed analogous searches in the control, non-tagged Rpo41p pull-down samples and found no matches. The results from these control experiments give confidence that the occurrence of the proteins that we detected in the mtRNAP–TAP pull-down is due to their specific interaction with the mt transcription complex. As noted below, their presence is consistent with previous biochemical and genetic findings.

### Mss116p inhibits mtRNAP activity *in vitro*

**Figure 4:**
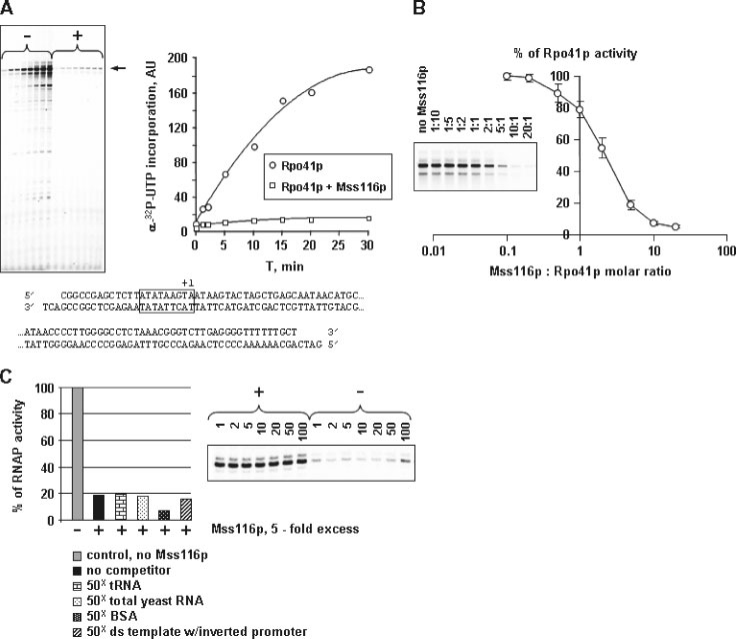
Mss116p inhibits transcription by mtRNAP. (A) Effect of Mss116p on run-off transcription by Rpo41p. A double-stranded DNA template containing a consensus Rpo41p promoter (boxed, transcription start site is at + 1, A) was transcribed in the presence (+) or absence (−) of a five-fold molar excess of Mss116p over Rpo41p. Run-off products accumulated at each time point were resolved by electrophoresis in a 15% polyacrylamide gel and quantified by PhosphorImager analysis (shown on graph); arrow indicates position of expected 77 nt run-off product. (B) Dose-dependence of inhibition. Transcription was carried out in the presence of increasing amounts of Mss116p for 15 min, and the products were analysed as described above. Transcription activity is expressed relative to the level observed in the absence of Mss116p. Error bars represent standard error for run-off transcripts (*n* = 10). (C) Effects of exogenous RNA, DNA or protein. Transcription was carried out in the presence or absence of a five-fold molar excess of Mss116p for 15 min in the presence or absence of a 50-fold excess of non-specific analogues of transcription complex components (DNA, RNA, protein) or of an RNA with a secondary structure (tRNA), as noted. The level of inhibition by Mss116p in the presence of each competitor is indicated as a percentage of the activity observed in the absence of Mss116p (control, 100%). The presence of competitor alone had no effect on RNAP activity, as shown for tRNA in the right panel

One of the most abundant proteins identified in the mtRNAP–TAP pull-down was a DEAD-box protein, Mss116p, which has previously been shown to be involved in mtRNA processing (Huang *et al.*, 2005; Seraphin *et al.*, 1989). However, in addition to these functions, there were also indications that Mss116p may be involved in the regulation of mt transcription (see below). As Mss116p has been purified and characterized (Del Campo *et al.*, 2007; Halls *et al.*, 2007), we elected to determine the effects of this protein in a purified mtRNAP transcription system. As shown in Figure 4A, B, Mss116p is a potent inhibitor of yeast mtRNAP in a steady-state transcription reaction, exerting an inhibitory effect of about 50% at a 2 : 1 Mss116p : Rpo41p molar ratio. This is not due to non-specific RNA- or DNA-binding activity or to contaminating nucleases, as a high excess of exogenous RNA or DNA did not prevent its inhibitory effect (Figure 4C). Furthermore, Mss116p had no effect on transcription by the multi-subunit RNAP from *Escherichia coli* (data not shown). Experiments to determine the mechanism of action of Mss116p on mtRNAP activity are under way.

## Discussion

Using a modified TAP protocol that was optimized for the isolation of mt proteins and complexes, we confirmed the association of yeast mtRNAP with proteins that had previously been suggested to be part of the mt transcription apparatus by genetic and biochemical studies (e.g. Mtf1p, Mtf2p, Sls1p, Pet309p). More importantly, our data identified a limited set of proteins that were not previously thought to be involved in interaction with the mt transcription complex, even though they had been implicated in mt gene expression by biochemical and genetic experiments (see Table 2). The potential roles of these proteins in mt transcription are discussed individually below.

Mss116p belongs to the DEAD-box protein family, members of which function in RNA and ribonucleoprotein particles (RNPs) structural rearrangements in all organisms (Cordin *et al.*, 2006; Linder, 2006). Previous studies have shown that Mss116p functions as a general RNA chaperone, which promotes the splicing of group I and group II mt introns, pre-mtRNA processing and mt translation (Halls *et al.*, 2007; Huang *et al.*, 2005). It has been proposed that Mss116p may carry out additional functions, similar to other multifunctional DEAD-box proteins (Fuller-Pace, 2006).

In accord with the notion that Mss116p may have alternative, possibly transcription-related functions, overexpression of Mss116p was found to suppress a deletion of the *SUV3* gene, which encodes the helicase component of the mt degradosome (Minczuk *et al.*, 2002). While this could have resulted from compensation for loss of the Suv3p helicase function by that of Mss116p, the non-processive RNA-unwinding activity of Mss116p and other DEAD-box proteins is expected to poorly substitute for that of a translocating RNA helicase such as Suv3p (Del Campo *et al.*, in press; Yang *et al.*, 2007). Further, it has been shown that the *SUV3* deletion may be independently suppressed by loss-of-function mutations in *RPO41* and *MTF1*, which inhibit mt transcription (Rogowska *et al.*, 2006). As the processes of RNA synthesis and RNA surveillance are likely to be coordinated, the latter observations were taken to suggest that down-mutations in *RPO41* and *MTF1* help to restore the balance in *SUV3*-deficient cells (Rogowska *et al.*, 2006). Based on the observation that overexpression of Mss116p also suppresses the *SUV3* deletion, and our finding that Mss116p is associated with mt transcription complex, we speculate that Mss116p may modulate the activity of mtRNA polymerase in a more direct manner, thereby synchronizing the rate of transcription with RNA degradation. Supporting this hypothesis, we have found that purified Mss116p is a potent inhibitor of transcription by yeast mtRNAP in a steady-state reaction (Figure 4). Association of Mss116p with the mt transcription complex may also facilitate RNA folding and RNP assembly on nascent transcripts through its RNA chaperone activity.

Another protein that we identified in the TAP–mtRNAP pull-downs, Pet127p, may also be involved in transcription regulation. As is the case for Mss116p, overexpression of Pet127p also suppresses deletion of the *SUV3* gene (Wegierski *et al.*, 1998). Moreover, overexpression of Pet127p suppresses the deletion of *DSS1*, which encodes the exonuclease component of the mt degradosome (Dziembowski *et al.*, 2003) and results in overall downregulation of mt transcription (Wegierski *et al.*, 1998). A potential mechanism for Pet127p action involving transcription termination/attenuation derives from observations that deletions of *PET127* suppress polar effects in certain mt transcription units (Chen *et al.*, 1999). These effects are manifested by a strong (∼40-fold) decrease in the abundance of transcripts from regions that lie downstream from the promoter (as compared to promoter-proximal regions) and appear to involve a conserved intergenic GC-rich stem–loop element that separates the differentially expressed parts of the transcription unit, as deletion of this element also suppresses the polar effect (Berke and Krause, 2004; Krause and Dieckmann, 2004). Transcription units in which this phenomenon has been observed usually encode a tRNA in the upstream region and a structural gene downstream. To account for the role of Pet127p in the differential abundance of upstream and downstream transcripts, it has been proposed that Pet127p causes termination/attenuation of transcription at the conserved element or, alternatively, binds to the GC-rich stem–loop in the RNA and blocks degradation of the upstream part of the transcript by a mt degradosome with 3′ to 5′ processivity (Fekete *et al.*, 2008). Further experiments to examine the role of Pet127p in transcription are under way.

Regarding the observed association of ribosomal proteins Yml6p and MrpL4p with yeast mtRNAP, it has recently been shown that human ribosomal protein L12 interacts with human mtRNAP and stimulates mt transcription *in vitro*, suggesting that L12 may serve to couple mt transcription and translation (Wang *et al.*, 2007). In our studies, we did not observe an interaction of the yeast homologue of human L12 (Mnp1p) with the mtRNAP. Instead, we observed the presence of two other ribosomal proteins, Yml6p and MrpL4p, which, like human L12, are part of the large ribosomal subunit. It is possible that Yml6p and MrpL4p perform a role similar to human L12 but are specific for yeast mtRNAP. In support of this, recent studies have found that purified yeast mt MrpL12p had no stimulatory effect on transcription in a human *in vitro* system and purified human L12 had no effect in a yeast mt transcription system (Temiakov *et al.*, unpublished). To explore this further, it will be necessary to purify Yml6p and MrpL4p and examine their effects in a yeast mt transcription system.

Some of the proteins that we identified in the TAP–mtRNAP pull-downs may have been recovered due to their association with the mt nucleoids, rather than directly with Rpo41p. For example, Hsp-like proteins have previously been shown to be a major component of human mt nucleoids (Wang and Bogenhagen, 2006), and consequently the presence of chaperone Hsp60p and chaperone-like protein Ssc1p in our pull-down may not be surprising.

In the same regard, Mgm101p has been found to be an integral nucleoid component that binds DNA and is required for repair of oxidatively damaged DNA (Meeusen *et al.*, 1999). As transcription complexes contain DNA and RNA, Mgm101p may have been pulled down through DNA–or RNA–protein interactions that are not directly related to Rpo41p. Similarly, Hsp60p has been shown to have DNA-binding activity (Kaufman *et al.*, 2003), and thus its presence in the pull-down may also be due to Rpo41p-associated DNA. Some investigators have suggested the use of DNAse and/or RNAse (Bertwistle *et al.*, 2004) to minimize potential non-specific interactions. We did not employ nuclease treatments in our studies because nucleic acids are an integral part of the transcription complex, and such treatments might disrupt or prevent the detection of important interactions.

One of the proteins that we identified, Kar2p, had not previously been thought to be associated with mitochondria and is believed to reside in the endoplasmic reticulum (ER) and to act as a negative regulator of Ire1p in the ER stress response (Okamura *et al.*, 2000; Panzner *et al.*, 1995). However, two studies demonstrated that Kar2p may be associated with Sls1p, a chaperone-like mt protein that has been shown to interact with Rpo41p (Boisrame *et al.*, 1998; Kabani *et al.*, 2000), which could account for its presence in the TAP pull-downs. It thus appears that Kar2p may be present in both cytosolic and mitochondrial compartments. In this regard, we note that a recent report on the mammalian mt proteome indicated that ∼16% of the mt proteins identified were initially believed to be cytosolic and not previously known to be associated with mitochondria (Pagliarini *et al.*, 2008).

Sls1p and a number of other previously known Rpo41p-associated proteins were not identified during the initial analysis of the TAP–mtRNAP pull-down proteins, but were subsequently identified by an informed search that allowed the presence of two oxidized residues per detected peptide, or of peptides that have non-tryptic origins. Importantly, these proteins were not identified in an informed search of a control sample that utilized a non-TAP-tagged mtRNAP. The use of more stable reducing agents (e.g. TCEP) or oxygen-less conditions (e.g. argon-saturated buffers) in future experiments may enhance detection of proteins that are particularly sensitive to oxidation, although the oxidation of peptides during electrospray MS is difficult to avoid.

Only a small number of peptides were found in an informed search for Mtf1p. This may reflect the nature of the association of this protein with the transcription complex, which occurs only during initiation (Mangus *et al.*, 1994). This suggests the possibility that other proteins that are transiently associated with the transcription complexes may also be underrepresented in the TAP pull-down. The use of ‘zero-time’ protein–protein crosslinking methods *in organello* (e.g. formaldehyde treatment) that do not affect TAP pull-down (Cojocaru *et al.*, 2008) may help to preserve such interactions in future studies.

In conclusion, we have demonstrated the potential of using a TAP pull-down procedure that has been optimized for mitochondria for the characterization of mt transcription complexes. This approach, and additional modifications that we have suggested, may prove useful in the analysis of other mt complexes. In addition, scanning insertion mutagenesis of the mtRNAP has identified a number of sites that appear to tolerate substitutions and modifications. This information may prove useful in the subsequent manipulation and engineering of yeast mtRNAP, as well as mtRNAPs from other organisms.
